# Natural Killer Cell Therapy in Ovarian Cancer: From Preclinical Potentials to Clinical Applications and Combination Strategies

**DOI:** 10.7150/ijbs.130260

**Published:** 2026-03-17

**Authors:** Tian Chen, Jiaqi Lu, Haiou Liu

**Affiliations:** 1 Obstetrics & Gynecology Hospital of Fudan University, Shanghai Key Lab of Reproduction and Development, Shanghai Key Lab of Female Reproductive Endocrine Related Diseases, Shanghai, China.; 2 Department of Gynecology, Obstetrics and Gynecology Hospital, Fudan University, Shanghai, China.

**Keywords:** ovarian cancer, natural killer cells, immunotherapy, tumor microenvironment, clinical trials, chimeric antigen receptor

## Abstract

Ovarian cancer represents a leading cause of mortality among gynecologic cancers, with limited therapeutic options for advanced and recurrent disease, highlighting an urgent need for innovative immunotherapies. Natural Killer (NK) cells, functioning as crucial effector cells of the innate immune system, have been identified as promising candidates for ovarian cancer treatment. This review systematically examines the evolving landscape of NK cell-based therapies for ovarian cancer, spanning their preclinical rationale, clinical translation, and innovative combination strategies. Nevertheless, the highly immunosuppressive tumor microenvironment (TME) of ovarian cancer and barriers to tumor infiltration pose significant challenges to their clinical efficacy. Here, we discuss various therapeutic strategies (such as cytokine-based agents, NK cell engagers and chimeric antigen receptor NK cells) designed to enhance NK cell activity, which leverage the unique characteristics of NK cells to complement standard treatments and potentiate combination immunotherapies. Ongoing preclinical and clinical investigations are paramount to converting these advances into efficacious therapies, ultimately revolutionizing the therapeutic paradigm for ovarian cancer.

## 1. Introduction

Ovarian cancer, as one of the common malignant tumors of the female reproductive system, presents a significant global clinical challenge due to its high mortality and recurrence rates 1, 2. Currently, first-line treatment for ovarian cancer is based on cytoreductive surgery combined with platinum-taxane chemotherapy. However, as most patients are diagnosed at an advanced stage and frequently develop chemoresistance 3, the efficacy of these conventional therapies is significantly limited, resulting in an unfavorable prognosis. Therefore, innovative therapeutic strategies are urgently required to improve the outcomes in ovarian cancer patients. In recent years, immunotherapy has made breakthrough progress in ovarian cancer treatment, and with ongoing characterization of its intricate tumor microenvironment 4, 5, it has become clear that overcoming the highly immunosuppressive tumor microenvironment of ovarian cancer will greatly enhance the efficacy of immunotherapy.

Within this context, natural killer (NK) cells, as pivotal effector cells of the innate immune system, have attracted considerable interest owing to their distinct anti-tumor potential 6. NK cells have the capability to recognize tumor cells without prior sensitization 7, and they play a dual role in synergistic tumor lysis and initiation of adaptive immunity by secreting various cytokines and chemokines. Preliminary evidence indicates a positive correlation between NK cell infiltration levels in various solid tumors and favorable patient prognoses 8, 9. Nevertheless, in the highly complex ovarian cancer tumor microenvironment (TME), infiltrating NK cells are often dysfunctional. This phenomenon highlights the substantial and yet untapped potential of NK cells in ovarian cancer immunotherapy 10.

This review aims to explore the cytotoxic effects and immunomodulatory functions of NK cells in anti-tumor immunity. Focusing on preclinical studies and evaluating the prospects of NK cell-based immunotherapeutic approaches in combined therapies for ovarian cancer, we present a summary of the research advancements and future directions of NK cells in this field. This work aims to offer a conceptual foundation for clinical application, ultimately bettering patient outcomes.

## 2. The antitumor functions of NK cells

Natural killer cells were described about 50 years ago as a distinct lymphoid cell subset, characterized by their lack of antigen-specific receptors and ability to rapidly eliminate virus-infected and malignant cells. Traditionally, NK cell subsets are classified based on the surface expression of CD56 and CD16a, predominantly into CD56^bright^CD16^lo^ NK cells and CD56^dim^CD16^hi^ NK cells 11. Recent advancements in high-dimensional single-cell analysis technologies have greatly deepened our comprehension of human NK cell heterogeneity 12-14. Researchers have categorized peripheral blood NK cells into three core subsets (NK1, NK2 and NK3), which are respectively enriched with classical CD56^dim^ NK cells, CD56^bright^ NK cells, and human cytomegalovirus-driven adaptive NK cells. The gene signatures of these NK subsets are applicable not only for classifying peripheral blood NK cells in healthy individuals but also for describing NK cell heterogeneity across diverse tissues. Notably, NK2 cells are found to be enriched in numerous tumor types, including ovarian cancer. While the features of NK cell dysfunction within the TME have been well established 15, the specific phenotype of these dysfunctional NK cells remains uncleared. The impaired cytotoxic capacity of NK cells in the TME correlates with the lower expression of effector molecules characteristic of NK2 cells, suggesting that an increased proportion of NK2 cells may be related to tumor immune escape.

### 2.1 Mediating direct cytotoxicity

**Figure 1:**
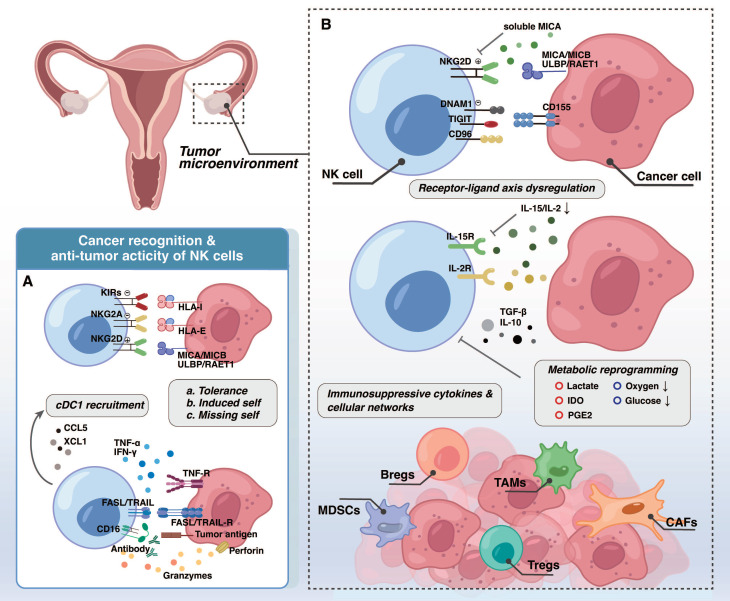
** Natural killer cell anti-tumor activity and TME-mediated suppression in ovarian cancer. (A)** Upon recognition, NK cells deploy multiplex cytotoxic mechanisms: 1) direct killing via perforin/granzyme release and death receptor pathways (e.g., FAS/FASL, TRAIL); 2) ADCC mediated by CD16a; and 3) immunomodulation via cytokine/chemokine secretion (e.g., IFN-γ, TNF-α, CCL5). Together, these functions coordinate a potent anti-tumor immune response. **(B)** The immunosuppressive TME compromises NK cell activity through three principal mechanisms: 1) dysregulated receptor-ligand interactions that favor inhibitory signaling and impair cancer cell recognition; 2) metabolic molecules (e.g., IDO, lactate) and soluble inhibitory factors (e.g., TGF-β, IL-10), leading to functional exhaustion; and 3) recruitment and activation of immunosuppressive cells, including TAMs, MDSCs, CAFs, Bregs, and Tregs.

The precise regulation of NK cell function relies on the dynamic balance between its surface activating and inhibitory receptors, engaging with specific ligands on target cells 16. Employing the classic "missing-self" mechanism, NK cells effectively distinguish "self" from "non-self." Their inhibitory receptors (e.g., KIRs, NKG2A heterodimer), recognize major histocompatibility complex class I (MHC-I) molecules on normal cells, thereby transmitting inhibitory signals that prevent attacks on normal cells (Figure 1). To evade T cell surveillance, malignant cells often downregulate MHC-I expression. This reduction inherently diminishes the inhibitory signals to NK cells, consequently leading to their activation. Ideally, this process enables NK cells to effectively identify tumor cells with absent or low MHC-I expression 17. For example, certain poorly differentiated ovarian cancer cell lines (OVCAR8 and CaOV3) remain considerable susceptibility to NK cell-mediated cytotoxicity despite the diminished MHC-I expression and high invasiveness 18.

Furthermore, NK cells express a variety of activating receptors, including NKG2D, natural cytotoxicity receptors (NCRs), and CD16 (FcγRIIIA). These receptors recognize upregulated stress ligands (e.g., MICA/B, B7-H6) on tumor cells, transmitting strong activating signals. It is noteworthy that the binding affinity of CD16a to the Fc region of an antibody is a key determinant of antibody-dependent cellular cytotoxicity (ADCC) efficiency 19, 20, resulting in effective tumor lysis. When activating signals surpass inhibitory signals, NK cells achieve full activation, and the specific mechanisms by which their different subsets induce tumor cell apoptosis vary slightly. The NK1 subset, characterized by high cytotoxicity, relies on the classic perforin/granzyme pathway to directly lyse target cells, while also expressing FasL to enhance killing effects 14. In contrast, NK2 cells highly express TRAIL (TNFSF10) to induce tumor cell apoptosis and predominantly engage in immunomodulation by secreting cytokines, consequently bolstering adaptive immune responses to synergistically achieve broader immune surveillance and defense 14.

### 2.2 Orchestrating immunomodulation

Besides direct cytotoxicity, NK cells play a significant immunomodulatory role in anti-tumor immunity. Their crosstalk with conventional type 1 dendritic cells (cDC1) cells exemplifies this interaction. Latest research highlights that the NK-cDC1 axis plays a crucial role in early immune surveillance of cells with chromosomal instability (CIN) or TP53 mutations, during the very early stages of high-grade serous ovarian carcinoma (HGSOC) development 21. It is known that activated NK cells efficiently recruit cDC1s into tumors by secreting chemokine (C-C motif) ligand 5 (CCL5) and XCL1 22, and continuously provide FLT3LG to maintain cDC1 proliferation and survival 23, 24. Studies demonstrate that NK cell-derived FLT3L is vital for maintaining cDC1 cell abundance, which correlates with improved overall survival (OS) and enhanced response to anti-PD-1 immunotherapy in melanoma patients 23. Moreover, NK cells secrete cytokines such as interferon-γ (IFN-γ), tumor necrosis factor-α (TNF-α), and granulocyte-macrophage colony-stimulating factor (GM-CSF), which together with CD40/ CD40 ligand (CD40L) interactions promote cDC1 maturation 25, 26, thereby enhancing their capacity to cross-present tumor antigens and activate CD8⁺ T cells.

Research further indicates that CD103^+^ Batf3-dependent cDC1 cells secrete interleukin-12 (IL-12) to stimulate NK cell production of IFN-γ, an interaction critical for suppressing tumor metastasis 27. One reported case of stage IV metastatic ovarian cancer showed positive outcomes, including tumor reduction, ascites regression, and declining tumor markers, after treatment with chemotherapy combined with a Wilms' tumor antigen 1 (WT1) dendritic cell vaccine, highly activated NK cell therapy, and Nivolumab 28. This indicates that combining multiple immunotherapies can elicit synergistic anti-tumor effects via a bidirectional activation mechanism between NK cells and dendritic cells, thus offering novel therapeutic avenues for patients with advanced cancer.

## 3. Impaired NK cell function in the ovarian cancer TME

Even with increasing clarity regarding the heterogeneity of NK cell subsets within the ovarian cancer TME, their intrinsic anti-tumor activity remains significantly constrained 9. This prevalent NK cell dysfunction is attributable to multiple convergent factors within the complex microenvironment. Firstly, dysregulation of NK cell receptor-ligand interactions directly impairs tumor cell recognition by NK cells. In addition, immunosuppressive cytokines and cellular networks, along with metabolic reprogramming within the TME, collectively impose significant constraints on NK cell survival and function.

### 3.1 Dysregulation of NK cell receptor-ligand interactions

Studies on the immunosuppressive state in individuals with primary and recurrent epithelial ovarian cancer highlight a downregulation of NKG2D 29, the crucial activating receptor on CD56^bright^ NK cells, primarily driven by abnormally elevated levels of soluble MICA (sMICA). MICA/B, which are MHC class I-related stress-induced antigens, typically activate NK cell anti-tumor immunity by engaging NKG2D 30. Research indicates that elevated concentration of sMICA in the serum competitively bind to NKG2D receptors on NK cells, thereby impeding NKG2D from effectively recognizing MICA/B ligands on tumor cells 29. Moreover, continuous exposure to such high sMICA levels can diminish NKG2D^+^ immune cell subsets and induce NK cell anergy 9, 29, facilitating immune escape.

NK cells extracted from the ascites of ovarian cancer patients show reduced expression of DNAM-1 (CD226), while inhibitory receptors TIGIT and CD96 are highly expressed 31. CD155 acts as a ligand for DNAM-1, TIGIT, and CD96, and in ovarian cancer, CD155 exhibits substantially higher affinity for TIGIT than for DNAM-1, thereby tilting the functional balance toward inhibition. Investigations have found that focal adhesion kinase (FAK) activity in high-grade serous ovarian cancer affects the expression of CD155 on tumor cells, and FAK inhibitors can mitigate the immunosuppressive effects of the CD155/TIGIT axis 32.

Therefore, upregulating activating receptors on NK cells 33, 34 and blocking inhibitory receptor signaling represent crucial avenues for developing innovative therapeutic strategies against ovarian cancer. Given the high proportion of PD-1^+^ NK cells also found in the peripheral blood and ascites of ovarian cancer patients 35, this provides a theoretical basis for further combining immune checkpoint inhibitors 36 to restore NK cell cytotoxicity, with related phase I/II clinical trials (NCT02671435, NCT02459301) have already been initiated in solid tumors.

### 3.2 Immunosuppressive cytokines and cellular networks

Malignant ascites (MA) develops in up to 90% of stage III/IV ovarian cancer patients, representing a critical pathological feature of advanced ovarian cancer 37. This intricate peritoneal fluid, rich in both cellular and acellular components 38, serves as a vital source for characterizing the ovarian cancer TME. Its fluctuating cytokine levels and heterogeneity of immune cell subsets are strongly correlated with adverse patient outcomes 37.

It is noteworthy that among multiple anatomical sites in advanced ovarian cancer patients, NK cells in the ascites represent the largest proportion of CD45^+^ leukocytes 39. Despite this numerical dominance, it fails to confer effective anti-tumor activity and are instead characterized by severely impaired function. This dysfunction results from the concerted action of various factors, including the abnormally elevated levels of cytokines such as TGF-β, certain subset of interleukins (IL), prostaglandin E2 (PGE2), and vascular endothelial growth factor (VEGF) 40, as well as the infiltration of immunosuppressive cell populations such as myeloid-derived suppressor cells (MDSCs) and regulatory T cells (Tregs) along with their inhibitory secretions 40, 41.

Among these factors, TGF-β has been widely documented for its inhibitory effect on NK cells 42, 43. Elevated TGF-β1 levels in ovarian cancer ascites correlate with NK cell dysfunction and reduced patient progression-free survival (PFS) and OS 44. Additionally, NK cells differentiated from umbilical cord blood CD34^+^ hematopoietic progenitor cells (HPC-NK) exhibit greater resistance to ascites-mediated suppression than peripheral blood NK cells (PB-NK) from healthy donors, suggesting the therapeutic potential of HPC-NK in adoptive cell therapy. Building on these observations, researchers confirmed that knockout of SMAD4, a key transcriptional regulator in the TGFβ signaling pathway, enables NK cells to maintain potent anti-tumor activity in TGFβ-enriched TME 45, and is applicable to various NK cell-based platforms such as anti-CD19-CAR-NK, HPC-NK (GTA002), and adaptive NK cell products (ADAPT-NK). Moreover, the bifunctional fusion protein Bintrafusp Alfa (BA), which simultaneously targets TGF-β and PD-L1, effectively clears TGF-β and VEGF from ascites and enhances cytolytic NK cell activity in ovarian cancer preclinical models 43, supporting BA as a promising new immunotherapeutic strategy for ovarian cancer.

PGE2 is identified as an immunomodulatory molecule synthesized by tumor cells via COX-1/COX-2, binds to EP2 and EP4 receptors on NK cells and directly impairing NK cell early tumor infiltration, intercellular contact, and IFN-γ production. This disruption hinders NK cell-driven polarization of tumor-associated macrophages (TAMs) toward classical inflammatory phenotype and CD8^+^ T cell activation 46. Crucially, the accumulation of immunosuppressive cells such as TAMs, CAFs, and MDSCs 47 within the TME establishes intricate crosstalk networks that synergistically impact NK cells, ultimately leading to persistent NK cell dysfunction 48, 49. Recent research has focused on a 'decoy' mechanism between CAFs and NK cells, in which CAFs continuously engage NK cells by upregulating multiple ligands for activating receptors NKG2D and DNAM-1, such as NECTIN2, PVR/CD155, and RAE-1. This interaction triggers the internalization and downregulation of activating receptors on NK cells, markedly impairing their capacity to recognize and eliminate cancer cells 50. Another recent study revealed that in a pancreatic cancer model, nociceptor neurons release calcitonin gene-related peptide (CGRP), which binds to receptor activity-modifying protein 1 (RAMP1) on CAFs, inhibiting IL-15 secretion by CAFs. This suppression impairs NK cell infiltration and cytotoxicity, thereby promoting tumor progression and cancer pain 51. The FDA-approved CGRP receptor antagonist Rimegepant reversed this effect 51, offering a paradigm for similar mechanistic investigations in ovarian cancer 52.

Collectively, these findings emphasize that a comprehensive understanding of how immunosuppressive cytokines and related cellular networks synergistically suppress NK cells in ovarian cancer TME, along with elucidation of the underlying mechanisms, holds significant translational value for developing novel therapeutic strategies.

### 3.3 Metabolic reprogramming

Tumor cells undergo significant metabolic reprogramming to meet their rapid proliferation and survival needs, and among all identified cancer metabolic reprogramming patterns, the Warburg effect is undoubtedly the most representative 53. One of the most direct consequences of the Warburg effect is the accumulation of large amounts of lactate in the TME. Elevated lactate levels in the TME directly impede NK cell cytotoxicity by inducing lysine lactylation (Kla) or indirectly by increasing the abundance of MDSCs 54. Furthermore, lactate accumulation is a critical factor that transcriptionally suppresses NAMPT expression in NK cells, leading to NAD⁺ metabolic imbalance and ultimately inhibiting their anti-tumor efficacy 55.

A common metabolic alteration in the TME is lipid accumulation, which is strongly associated with immune dysfunction. Recent investigations have revealed that NK cells massively uptake polar lipids, particularly phosphatidylcholine PC(36:1), from ovarian cancer ascites through the scavenger receptor SR-B1 39. This aberrant lipid accumulation compromises the integrity of NK cell plasma membranes and interferes with the reduction of neutral lipid droplet levels, subsequently damages mitochondrial function, resulted in a comprehensive impairment of cytotoxic function. Pharmacological blockade of SR-B1 or lipid depletion approaches can partially ameliorate these metabolic deficiencies 39, underscoring the potential therapeutic utility of targeting lipid metabolism in NK cell immunotherapy.

Indoleamine 2,3-dioxygenase (IDO), the rate-limiting enzyme in tryptophan metabolism, produces kynurenine (KYN), which is often elevated in the TME of various tumors. Research suggests that Kyn activates aryl hydrocarbon receptor (AhR), a transcription factor that binds to the xenobiotic-responsive element (XRE) site on the ADAM10 promoter, thereby upregulating ADAM10 transcription and expression. This process enhances the shedding of NKG2D ligands (NKG2DLs) from tumor cell, reducing membrane-bound NKG2DLs while increasing soluble NKG2DL release 56. Consequently, this impairs NK cell recognition and binding of tumor cells via NKG2D receptors 56. Kyn can also induce ferroptosis in NK cells through AhR-independent pathways 57, substantially aggravating both the depletion of NK cells and their functional impairment within the TME. While several IDO1 inhibitors have advanced to clinical trials, results from a phase III trial (ECHO-301/KEYNOTE-252) showed no additional clinical benefit when combined with anti-PD-1 therapy. However, researchers have since developed NTRC 3883-0, an IDO1 inhibitor capable of inhibiting tryptophan metabolism in interferon-γ (IFN-γ)-stimulated primary ovarian cancer cells 58.

Recent proteomic analyses of ovarian cancer ascites have revealed that approximately 50% of patients exhibit higher total iron levels in ascites than in healthy human serum 59, suggesting that ascites may serve as a localized iron source for iron-dependent ovarian cancer cells. Further research has confirmed that the FDA-approved iron chelator deferoxamine alters mitochondrial integrity, leading to the release of mtDNA into cytoplasm, activation of the cGAS-STING-IRF3 pathway, and increased type I interferon (IFN-I) release. This cascade subsequently promotes IL-15 secretion by tumor-associated dendritic cells (tDCs), thereby enhancing NK cell infiltration and cytotoxic function 59. These findings strongly demonstrate that elucidating and targeting the metabolic dysregulation features of ovarian cancer represents a viable strategy to reverse immunosuppression and unleash the potential of NK cell-based immunity.

Researchers have implemented targeted metabolic reprogramming of NK cells during *ex vivo* expansion to foster intrinsic resilience against metabolic suppression within the TME. A primary exemplar of this strategy is METR-NK, a metabolically programmed NK cell product that integrates specific metabolic modulators during manufacturing to delay cellular senescence and bolster cytotoxicity. Currently, METR-NK is being evaluated for advanced epithelial ovarian cancer both as a neoadjuvant intraperitoneal therapy (NCT06395844) and in combination with Solantinib (NCT06884345), to leverage the therapeutic synergy between metabolic fitness and anti-angiogenic targeting.

## 4. Therapeutic strategies to enhance NK cell function

Adoptive cell therapy (ACT), which involves ex-vivo selection, activation, and large-scale expansion of immune effector cells with anti-tumor activity followed by reinfusion into the patient, has emerged as a major strategy in oncology. Among these, NK cells are regarded as highly promising effector cells due to their antigen-independent recognition, broad target range, and low risk of graft-versus-host disease (GVHD), showing potential in both hematologic malignancies and solid tumors. Several clinical trials have recently assessed the safety and efficacy of NK cell-based ACT in ovarian cancer. (Table 1).

### 4.1 Cytokine-based agents

As a direct and efficient strategy, cytokine-based agents can bypass the complex immunosuppressive network within the TME and rapidly activate NK cell cytotoxicity through exogenous delivery of high-concentration cytokines. This approach is especially suitable for the CD56^bright^ NK cell subset, which expresses high levels of cytokine receptors 14. A primary objective of current research is to generate CIML NK cells through in-vitro activation with IL-2, IL-15, and IL-18 60, 61. These cells exhibit enhanced IFN-γ production and cytotoxicity against ovarian cancer 61. A study combining assay for transposase-accessible chromatin sequencing (ATAC-seq), cellular indexing of transcriptomes and epitopes by sequencing (CITE-seq), and single-cell RNA sequencing (scRNA-seq) revealed that IL-12/15/18 rapidly induces epigenetic remodeling in human NK cells, forming enriched memory-like (eML) NK cells and effector conventional NK (effcNK) cells 62, with significantly increased chromatin accessibility in the IFNG promoter region of eML NK cells. This work advances our understanding of CIML NK cell heterogeneity and suggests that personalized immunotherapeutic strategies could be developed based on their distinct phenotypes in the future. Preliminary findings from an investigator-initiated Phase 1b trial (NCT06321484) evaluated the safety and tolerability of intraperitoneally (i.p.) delivered autologous CIML-NK cells in patients with platinum-resistant ovarian cancer (PROC) 63. Initial results from the first two patients demonstrated that autologous CIML-NK products of sufficient cell number were successfully generated following a 6-day culture in IL-2-supplemented media, and their i.p. administration was well-tolerated with no reported adverse events.

**Figure 2:**
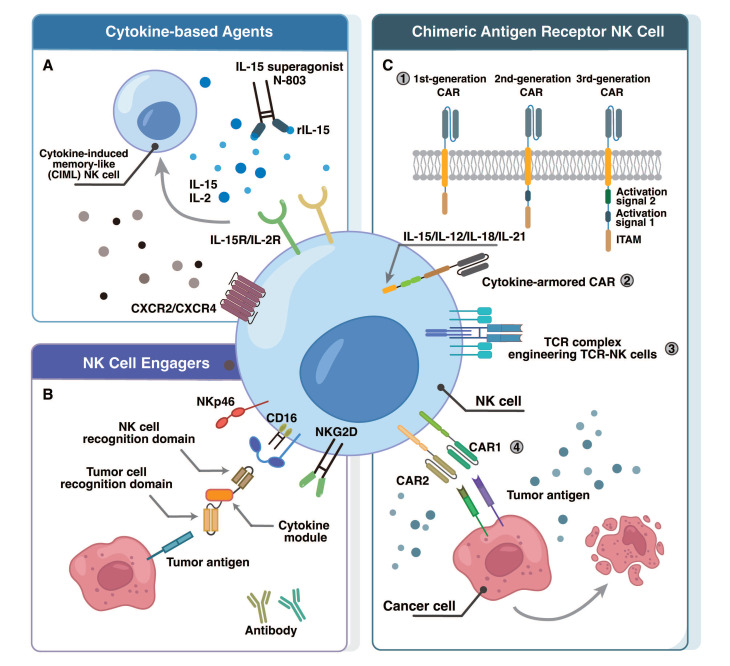
** Potential strategies for enhancing NK cell therapy in ovarian cancer. (A)** Cytokine Modulation for NK Cell Enhancement: This strategy leverages specific cytokines and chemokine receptor engineering (e.g., N-803, CIML-NK cell) to boost NK cell activity, proliferation, and tumor targeting. Engineering NK cells with chemokine receptors like CXCR2/CXCR4 improves their migration and infiltration into tumor sites. **(B)** NKCEs are engineered to guide NK cells to tumor cells, establishing an immune complex by engaging both tumor-specific antigens and NK cell activating receptors (e.g., CD16, NKG2D, NKp46). **(C)** Chimeric antigen receptor NK cells: 1) CAR design has evolved significantly: first-generation CARs feature an extracellular antigen-binding domain (scFv) linked to an intracellular CD3ζ signaling domain; second-generation CARs incorporate an additional costimulatory domain (e.g., CD28, 4-1BB) to CD3ζ for improved activation and persistence; third-generation CARs further enhance signaling by including two costimulatory domains. 2) To boost their fitness and longevity in the tumor microenvironment, NK cells can be armored with cytokines. 3) Other strategies like TCR-complex engineering can enable recognition of intracellular tumor peptides or HLA-independent antigens, respectively. 4) Advanced CAR designs facilitate multi-antigen targeting to enhance tumor coverage and mitigate antigen escape.

Adoptive therapies based on allogeneic cytokine-activated NK cells are being investigated clinically (Figure 2). The IL-15 superagonist N-803, which combines IL-15 with an activating mutation, an IL-15Rα sushi domain for trans-presentation, and an IgG1-Fc fragment to extend its half-life, overcomes the short half-life limitation of conventional cytokines 64, 65. It stimulates HPC-NK cell expansion, IFN-γ secretion, and cytotoxicity against leukemia and ovarian cancer cells. Preliminary results from a phase II clinical trial (NCT03054909) have been reported for N-803 as maintenance therapy in advanced ovarian cancers. Preclinical investigations also suggest that combining N-803 with Avelumab significantly reduces ovarian cancer tumor burden 66.

Moreover, compared to IL-2, N-803 enhances NK cell and CD8⁺ T-cell function while avoiding the common IL-2-mediated expansion of Tregs. Another novel engineered IL-2 variant, Nemvaleukin alfa, was evaluated in the clinical trial ARTISTRY-1 (NCT02799095). It selectively binds to intermediate-affinity IL-2 receptors, preferentially activating and expanding NK cells and CD8⁺ T cells while minimizing Treg proliferation. With a low rate of treatment discontinuation due to treatment-related adverse events (TRAEs), it demonstrates a manageable safety profile 67. An exploratory analysis in the ARTISTRY-1 study included 14 evaluable patients with platinum-resistant ovarian cancer who received nemvaleukin alfa combined with pembrolizumab, resulting in an objective response rate (ORR) of 21% 67. These findings prompted the launch of the phase III multicenter trial (ARTISTRY-7, NCT05092360), which will evaluate the efficacy of this combination compared to chemotherapy in patients with ovarian cancer.

Beyond standard cytokine priming, FATE-NK100 represents a novel adaptive NK cell therapy modulated with IL-15 and a glycogen synthase kinase 3β (GSK3β) inhibitor. In the phase I trial, nine patients were treated, and no dose-limiting toxicities (DLTs) were observed 68. Given the clinical benefit, three patients (33%) underwent retreatment, including one who achieved a partial remission (48% tumor reduction). Functional assays of *in vivo* samples confirmed that these FATE-NK100 cells persisted in the peritoneal cavity for up to 21 days, exhibiting superior* in vivo* function compared to the patient's endogenous NK cells (NCT03213964) 69.

### 4.2 NK cell engagers

From a long-term treatment perspective, cytokine-based agents may inevitably induce cytokine release syndrome (CRS) in the complex TME and have limited target specificity. In contrast, antibody-dependent NK Cell Engagers (NKCEs) can specifically recognize and bind to tumor-associated antigens (TAAs) on tumor cells and NK cell activating receptors, displaying activity only in the presence of tumor cells 70-72. Many NKCEs target CD16a on the NK cell, a receptor naturally responsible for recognizes antibody Fc fragments (Fcγ) and mediates ADCC. These NKCEs are therefore engineered to actively and efficiently bridge NK cells with tumor cells, achieving enhanced, redirected ADCC 73.

The conventional human epidermal growth factor receptor 2 (HER2)-targeting monoclonal antibody Trastuzumab has low affinity between its Fc region and the CD16a receptor on NK cells, and it readily cross-reacts with other Fcγ receptors (e.g., CD16b, CD32b), resulting in suboptimal ADCC effects. To overcome this limitation, researchers developed a novel bispecific killer cell engager, BiKE:HER2/CD16a (BiKE:E5C1), which uses high-affinity anti-CD16a and anti-HER2 VHH to precisely bridge NK cells to HER2^+^ ovarian cancer cells 74, 75. *In vitro*, BiKE:E5C1 enhanced NK cell activation, increasing the release of perforin, granzyme B (GZMB), TNF-α, and IFN-, and demonstrated approximately 100-fold greater potency than trastuzumab. In a humanized mouse model of ovarian cancer, BiKE-E5C1 combined with laNK92 cells eradicated metastatic HER2^+^ tumors 76. Another Tribody, [(HER2) ₂xCD16], which contains two HER2-binding domains, is capable of induce the lysis of HER2^+^ ovarian tumor cells even with minimal infiltration of NK and γδ T cells 77. Such a framework can be extended to the design of trispecific NK cell engagers (TriKEs), for example by co-targeting multiple activating receptors on NK cells or by fusing them with cytokines to achieve synergistic signal activation and functional support 78, 79.

A recent phase I study (NCT04259450) reported results for AFM24 that among 35 patients with advanced solid tumors, 10 (approximately 28.6%) achieved stable disease 80, which is a positive signal for advanced patients who have received multi-line therapy.

### 4.3 CAR-NK cells

While NKCEs efficiently augment ADCC by bridging TAAs to NK cell receptors, their overall efficacy is largely limited by tumor antigen density, heterogeneity, and the persistence of infused NK cells* in vivo*. Therefore, chimeric antigen receptor (CAR)-NK cells, which are genetically engineered to directly recognize tumor antigens, have emerged as a highly promising advancement in NK cell-based immunotherapy 81. CAR-NK therapy can utilize MHC-mismatched allogeneic NK cells, thereby reducing the risks of CRS and GVHD 82. Although most CAR-NK research remains in preclinical or early-phase clinical trials, their favorable safety and initial efficacy profiles have been widely recognized.

CAR-NK cell products derive from diverse sources, such as NK92 cell lines, induced pluripotent stem cell (iPSC)-derived NK cells, CD34⁺ hematopoietic stem and progenitor cells (HSPCs) from umbilical cord blood (CB), and peripheral blood-derived NK (PB-NK) cells. To facilitate "off-the-shelf" therapy, iPSC-derived NK cells are a major focus of current research 83-86. Beyond addressing the extended preparation times, high costs of autologous therapies, and the donor-to-donor variability of peripheral blood NK cells, the absence of DLTs in the first patient treated with GPC3-targeted iPSC-derived CAR-NK cells (jRCT2033200431) further supports the clinical feasibility of this standardized, mass-producible platform 87.

The design of CAR-NK cells builds on the framework established for CAR-T cells. Currently, second and third-generation CAR-NK cells, building on the first-generation CAR-NK cells that only contained a CD3ζ activation signal, additionally integrate costimulatory domains 88, 89, such as the CD28 costimulatory domain. This domain recruits the intrinsically expressed Src family kinases (e.g., LCK) via its PYAP motif, subsequently phosphorylating CD3ζ and activating ZAP70 to enhance NK activation signals 90. In ovarian cancer, an NK-CAR construct comprising NKG2D-2B4ζ expressed in iPSC-NK cells was shown to markedly improve cytotoxicity against antigen-positive ovarian cancer targets 91. Similarly, a third-generation CAR containing costimulatory domains (CD28 and 4-1BB) along with a CD3ζ signaling domain was introduced into NK-92 cells. *In vitro* assays confirmed that these engineered NK-92 cells exhibited significantly enhanced killing of CD24^high^ SKOV3 and OVCAR3 ovarian cancer cells 92.

Selection of appropriate tumor-associated antigens (TAAs) is critical to achieving precise targeting and minimizing off-tumor toxicity. Several TAAs are currently under investigation as CAR-NK targets in ovarian cancer, including mesothelin (MSLN) 93, 94, CD24 92, HER2 95, α-folate receptor (αFR) 96, Claudin-6 97, CD44 98, and glypican-3 (GPC3) 99.

Nevertheless, inadequate cytokine and chemokine support within the ovarian TME severely limits CAR-NK cell tumor infiltration and *in vivo* persistence, posing a major translational challenge for solid tumor therapy. Recent studies have explored strategies such as cytokine armoring and chemokine receptor engineering. A study revealed that in CAR-NK cells armed with engineered expression of the IL-2Rβγ agonist Neo-2/15 100, Neo-2/15 can continuously and stably activate the STAT5/Akt signaling pathway, driving persistent high expression of the transcription factor c-Myc. c-Myc directly upregulates glucose and glutamine transporter expression, enhancing the metabolic competitiveness of cells in nutrient-deprived TME, while activating the endoplasmic reticulum (ER) stress sensor IRE1α to promote XBP1s splicing and nuclear translocation. Nuclear XBP1s subsequently upregulates nuclear respiratory factor 1 (NRF1), maintaining mitochondrial integrity, upregulating anti-apoptotic proteins, and suppressing ER stress-induced apoptosis. Although the enhancement mechanism in Neo-2/15-armed CAR-NK cells differs from that in cytokine-pretreated MSLN-CAR CIML NK cells 93, both approaches similarly improve *in vivo* persistence and anti-tumor efficacy 93, 100. Another study focused on chemokine receptor engineering. Observing that IFN-γ and TNF-α produced during NK-92-αFR-CAR-mediated killing of ovarian cancer cells induce CXCL10 via JAK1/2-STAT1 and NF-κB pathways 96, researchers engineered NK-92 cells to co-express CXCR3A and αFR-CAR (termed NK-92-αFR-CAR-CXCR3A). This modification successfully enhanced the migration and tumor-infiltrating abilities of the CAR-NK cells 96.

The optimization of CAR-NK adoptive therapy has advanced through diverse strategies 81, including multi-antigen targeting, chemokine receptor engineering, cytokine armoring, transcriptional remodeling, and the development of TCR-engineered NK cells. Subsequent research will focus on identifying rational combinations and optimal timing for incorporating CAR-NK cell therapy with established or emerging treatments (e.g., oncolytic viruses 101, 102, chemotherapy, immune checkpoint inhibitors, or immunomodulatory drugs 103), to advance CAR-NK cell therapies for solid tumors into clinical practice.

## 5. Concluding remarks

NK cell therapy has demonstrated remarkable preclinical potential in ovarian cancer. As a central effector cell of innate immunity, NK cells can directly eliminate tumor cells and broadly enhance adaptive immune responses 21, 22. Therefore, therapeutic approaches that efficiently leverage these innate functions have emerged as crucial complements to conventional treatments and are vital for boosting the efficacy of combination immunotherapies. By combining NK cell-based strategies with chemotherapy, immune checkpoint inhibitors, or oncolytic viruses 104, thereby enhancing anti-tumor activity and *in vivo* persistence, they represent a transformative avenue for improving outcomes in ovarian cancer patients.

However, the clinical application of ovarian cancer NK cell therapies remains hindered by TME-mediated suppression of infiltration and activity, coupled with the donor heterogeneity and prohibitive costs of conventional cell sources. In light of this, the development of next-generation NK cell platforms is pivoting toward industrialization and precision, centered on iPSC technology and targeted gene editing. Through the establishment of master cell banks from a single, rigorously characterized clone, ensuring a uniform genetic background and consistent functional potency across production lots, the iPSC-derived NK cell platform provides the unparalleled scalability and clonal consistency required for standardized, off-the-shelf immunotherapy 105. Furthermore, leveraging the high gene-editing efficiency of iPSCs, researchers can precisely and stably engineer multiple functional enhancements. For instance, through the co-expressing of a high-affinity non-cleavable CD16a and a membrane-bound IL-15/IL-15R fusion protein, combined with the knockout of the CD38, triple gene-edited iPSC-derived NK cells (termed iADAPT NK) can persist and function *in vivo* in the absence of exogenous cytokine support 86. Alternatively, the deletion of the negative regulator CISH significantly enhances the sensitivity of iPSC-NK cells to low-dose IL-15 and promotes mTOR-mediated metabolic fitness, thereby substantially improving their* in vivo* persistence and anti-tumor activity 106.

In conclusion, future research should prioritize a comprehensive elucidation of the complex interactions between NK cells and the TME, employing multi-omics approaches such as single-cell transcriptomics, spatial transcriptomics, and metabolomics. This foundational insight will be critical for refining therapeutic target selection, optimizing *in vitro* expansion and *in vivo* delivery platforms, and advancing personalized combination approaches that integrate NK cell-based therapy with other modalities. By synthesizing current advances and delineating future directions, this review seeks to support and advance the refinement of more effective NK cell-based immunotherapies for ovarian cancer.

## Funding

This work was supported by the National Natural Science Foundation of China (82273205, 82203665, 82473274, 82503116, 82503386), Natural Science Foundation of Shanghai (23ZR1408300) and Shanghai Science and Technology Innovation Action Plan (23Y11909500, 23Y11901800).

## Figures and Tables

**Table 1:** Novel NK cell-based immunotherapy clinical trials for ovarian cancer.

NCT Number	Study Title	Phase (status)	Conditions	Interventions	Cell Source
**Non-genetically modified NK cells**					
NCT06321484	Intraperitoneal Cytokine-Induced Memory Like (CIML) Natural Killer (NK) Cells in Recurrent Ovarian Cancer	Phase I (Recruiting)	Platinum-resistant Ovarian Cancer Recurrent Ovary Cancer Ovarian Cancer	BIOLOGICAL: CIML NK Cells DRUG: IL-2	Peripheral Blood
NCT06710288	A Phase 2, Open-label, Single-arm Study of Autologous M-CENK Adoptive Cell Therapy And N-803 (IL-15 Superagonist) In Combination with Gemcitabine in Participants with Recurrent Platinum-Resistant High-Grade Ovarian Cancer	Phase II (Recruiting)	Platinum-resistant Ovarian Cancer	DRUG: Gemcitabine BIOLOGICAL: N-803 BIOLOGICAL: M-CENK	Peripheral Blood
NCT06395844	Safety and Efficacy of Intraperitoneal Injection of METR-NK Cells as Neoadjuvant Therapy for Advanced Epithelial Ovarian Cancer	Phase I /II (Recruiting)	Ovarian Cancer	DRUG: METR-NK Cells	Peripheral Blood
NCT06884345	METR-NK Cells in Combination with Anti-angiogenic Neoadjuvant Therapy for Advanced Epithelial Ovarian Cancer	Phase I /II (Active, not recruiting)	Ovarian Cancer	DRUG: METR-NK Cells combined with Solvatinib	Peripheral Blood
NCT03213964	Intraperitoneal Delivery of Adaptive Natural Killer (NK) Cells (FATE-NK100) With Intraperitoneal Int	Phase I (Completed)	Epithelial Ovarian Cancer Fallopian Tube Cancer Primary Peritoneal Cancer	BIOLOGICAL: FATE-NK100 DRUG: IL-2	Peripheral Blood
**Engineered NK cells**					
NCT06342986	Intraperitoneal FT536 in Recurrent Ovarian, Fallopian Tube, and Primary Peritoneal Cancer	Phase I (Recruiting)	Gynecologic Cancer Ovarian Cancer Fallopian Tube Cancer Primary Peritoneal Cavity Cancer	DRUG: FT536 DRUG: Fludarabine DRUG: CY	iPSC
NCT03692637	Study of Anti-Mesothelin Car NK Cells in Epithelial Ovarian Cancer	Early Phase I (Unknown)	Epithelial Ovarian Cancer	BIOLOGICAL: anti-Mesothelin CAR-NK Cells	Peripheral Blood
NCT05410717	CLDN6/GPC3/Mesothelin/AXL-CAR-NK Cell Therapy for Advanced Solid Tumors	Phase I (Recruiting)	Stage IV Ovarian Cancer Refractory Testis Cancer Recurrent Endometrial Cancer	BIOLOGICAL: Claudin6 CAR-NK cells BIOLOGICAL: GPC3 CAR-NK cells BIOLOGICAL: Mesothelin CAR-NK cells BIOLOGICAL: AXL CAR-NK cells	Peripheral Blood
NCT05856643	Single-arm, Open-label Clinical Study of SZ011 in the Treatment of Ovarian Epithelial Carcinoma	Early Phase I (Recruiting)	Epithelial Ovarian Cancer	DRUG: SZ011 CAR-NK	Unknown
NCT05776355	NKG2D CAR-NK & Ovarian Cancer	Unknown	Ovarian Cancer	Biological: NKG2D CAR-NK	Unknown
NCT05922930	Study of TROP2 CAR Engineered IL15-transduced Cord Blood-derived NK Cells Delivered Intraperitoneally for the Management of Platinum Resistant Ovarian Cancer, Mesonephric-like Adenocarcinoma, and Pancreatic Cancer	Phase I /II (Recruiting)	Ovarian Cancer Pancreatic Cancer Adenocarcinoma	DRUG: TROP2-CAR-NK DRUG: Cyclophosphamide DRUG: Fludarabine	Cord Blood

*Abbreviations:* CAR: Chimeric Antigen Receptor, CIML: Cytokine-Induced Memory Like, CLDN6: Claudin 6, CY: Cyclophosphamide, GPC3: Glypican-3, IL-15: Interleukin-15, IL-2: Interleukin-2, iPSC: Induced Pluripotent Stem Cells, METR: Metabolic Remodeling, NK: Natural Killer, NKG2D: Natural Killer Group 2D, TROP2: Trophoblast Cell-Surface Antigen 2.

## Abbreviations

ACT: Adoptive cell therapy
ADAPT-NK: adaptive natural killer cell products
ADCC: antibody-dependent cellular cytotoxicity
AhR: aryl hydrocarbon receptor
ATAC-seq: assay for transposase-accessible chromatin sequencing
BA: Bintrafusp Alfa
BiKE: bispecific killer cell engager
CAR: chimeric antigen receptor
CAR-NK: chimeric antigen receptor natural killer cells
CB: cord blood
CCL5: chemokine (C-C motif) ligand 5
CIML: cytokine-induced memory-like
CIN: chromosomal instability
CITE-seq: cellular indexing of transcriptomes and epitopes by sequencing
COX-1/COX-2: cyclooxygenase-1/-2
CRS: cytokine release syndrome
DLTs: dose-limiting toxicities
eML: enriched memory-like
effcNK: effector conventional natural killer cells
EGFR: epidermal growth factor receptor
FAK: focal adhesion kinase
FDA: Food and Drug Administration
GM-CSF: granulocyte-macrophage colony-stimulating factor
GPC3: glypican-3
GSK3β: glycogen synthase kinase 3β
GVHD: graft-versus-host disease
HCMV: human cytomegalovirus
HER2: human epidermal growth factor receptor 2
HGSOC: high-grade serous ovarian carcinoma
HPC-NK: hematopoietic progenitor cell-derived natural killer cells
HSPCs: hematopoietic stem and progenitor cells
IDO: indoleamine 2,3-dioxygenase
IFN-I: type I interferon
IFN-γ: interferon-γ
IL: interleukins
IL-12: interleukin-12
IL-15: interleukin-15
IL-18: interleukin-18
IL-2: interleukin-2
iPSC: induced pluripotent stem cell
KIRs: killer cell immunoglobulin-like receptors
Kla: lysine lactylation
KYN: kynurenine
MA: malignant ascites
MDSCs: myeloid-derived suppressor cells
MHC-I: major histocompatibility complex class I
MICA/B: MHC class I-related stress-induced antigens
MSLN: mesothelin
NCRs: natural cytotoxicity receptors
NKG2A: NK group 2 member A
NKG2D: NK group 2 member D
NKG2DLs: NKG2D ligands
NKCEs: NK Cell Engagers
NK cells: Natural Killer cells
ORR: objective response rate
OS: overall survival
PB-NK: peripheral blood natural killer
PD-L1: programmed cell death-ligand 1
PFS: progression-free survival
PGE2: prostaglandin E2
PROC: platinum-resistant ovarian cancer
RAMP1: receptor activity-modifying protein 1
scRNA-seq: single-cell RNA sequencing
SR-B1: scavenger receptor B1
TAAs: tumor-associated antigens
TAMs: tumor-associated macrophages
TGF-β: transforming growth factor-beta
TME: tumor microenvironment
TNF-α: tumor necrosis factor-α
TRAIL: TNF-related apoptosis-inducing ligand
TRAEs: treatment-related adverse events
Tregs: regulatory T cells
TriKEs: trispecific NK cell engagers
tDCs: tumor-associated dendritic cells
VEGF: vascular endothelial growth factor
WT1: Wilms' tumor antigen 1
XRE: xenobiotic-responsive element
αFR: α-folate receptor
